# Bacterial community assembly in activated sludge: mapping beta diversity across environmental variables

**DOI:** 10.1002/mbo3.388

**Published:** 2016-10-19

**Authors:** Siavash Isazadeh, Shameem Jauffur, Dominic Frigon

**Affiliations:** ^1^ Department of Civil Engineering and Applied MechanicsMcGill UniversityMontrealQuebecCanada; ^2^ Present address: Life Sciences GroupAir Liquide Delaware Research & Technology CenterNewarkUSA

**Keywords:** activated sludge, beta diversity, biosolids, environmental variables, ozone, population assembly

## Abstract

Effect of ecological variables on community assembly of heterotrophic bacteria at eight full‐scale and two pilot‐scale activated sludge wastewater treatment plants (AS‐WWTPs) were explored by pyrosequencing of 16S rRNA gene amplicons. In total, 39 samples covering a range of abiotic factors spread over space and time were analyzed. A core bacterial community of 24 families detected in at least six of the eight AS‐WWTPs was defined. In addition to the core families, plant‐specific families (observed at <50% AS‐WWTPs) were found to be also important in the community structure. Observed beta diversity was partitioned with respect to ecological variables. Specifically, the following variables were considered: influent wastewater characteristics, season (winter vs. summer), process operations (conventional, oxidation ditch, and sequence batch reactor), reactor sizes (pilot‐scale vs. full‐scale reactors), chemical stresses defined by ozonation of return activated sludge, interannual variation, and geographical locations. Among the assessed variables, influent wastewater characteristics and geographical locations contributed more in explaining the differences between AS‐WWTP bacterial communities with a maximum of approximately 26% of the observed variations. Partitioning of beta diversity is necessary to interpret the inherent variability in microbial community assembly and identify the driving forces at play in engineered microbial ecosystem.

## 1 Introduction

Activated sludge (AS) system employed in wastewater treatment plants (WWTPs) is among the world's largest biotechnological processes. Over the last century, AS process has experienced continuous modifications in design and operation to improve its efficiency (Seviour & Nielsen, 2010). At the heart of the AS processes, consortia of heterotrophic microorganisms transform incoming organic compounds into biomass and CO_2_ through specific metabolisms. Modifications of the process often aim at promoting control over specific eco‐physiological characteristics of the microbial community. Thus, rationally developing further process modifications require the recognition and understanding of the key abiotic factors influencing community assembly within AS systems. As such, quantifying how the variations in the environmental or operational variables influence the microbial community composition remains a fundamental goal of wastewater microbiology.

Advancements in molecular biology techniques used to study the diversity of 16S rRNA or functional genes (e.g., FISH, T‐RFLP, DGGE, and amplicon cloning) have provided new opportunities to better understand the complexity of microbial ecosystems. High‐throughput sequencing techniques have expanded considerably the depth of description of microbial diversities in wastewater treatments plants with AS process (AS‐WWTPs) (Pinto & Raskin, 2012). Yet, the challenge remains to ascertain the effect of abiotic variations (e.g., operational, spatial, and temporal) on the structure of the assembly of bacterial communities.

Several lines of evidence link bacterial community compositions and performance of biological wastewater treatment processes to operational variations such as: influent composition (Akarsubasi et al., 2005; Lee, Kim, Hwang, O'Flaherty, & Hwang, 2009), reactor configuration (Rowan et al., 2003), temperature variation (Siripong & Rittmann, 2007), and solids retention time (SRT) (Akarsubasi, Eyice, Miskin, Head, & Curtis, 2009). In addition, ecosystem size has been found to influence the observed distances in community composition (called beta diversity over time (Soininen, 2010), and reactor scale can affect species selection based on cellular morphology (Martins, Pagilla, Heijnen, & van Loosdrecht, 2004). In a comprehensive laboratory‐scale study, Pholchan, Baptista, Davenport, and Curtis (2010) found that the microbial community of AS reactors was affected by different operational conditions and reactor configurations; however, the relationship between the performance and community diversity was not explicitly associated. Zhang, Shao, and Ye (2012) studied the effects of geographical variation on population structure of AS‐WWTPs and demonstrated that some core genera were shared between samples in spite of large geographical distances. Valentín‐Vargas, Toro‐Labrador, and Massol‐Deyá (2012) monitored over 1 year the bacterial community compositions of two geographically distinct AS wastewater treatment systems of different sizes, and they determined that the largest bioreactor had a less dynamic composition. Although these studies highlighted the importance of environmental factors on bacterial community assembly, a systematic quantification of the abiotic parameters contribution on the species composition and distribution of bacterial communities remains a main interest in the applied microbiology.

To quantify the impact of the different environmental variables in determining bacterial community compositions, 39 biomass samples from eight full‐scale AS‐WWTPs and two pilot‐scales AS‐WWTPs were characterized using high‐throughput pyrosequencing of 16S rRNA gene amplicon. Observed variations in community composition (called beta diversity) were partitioned to obtain the relative magnitude of contribution for the selected environmental variables. First, eight full‐scale AS‐WWTPs were sampled in different seasons (during the same year) and then 4 years after to assess the relative stability of the community at one plant. Differences among the plants in influent characteristics, treatment processes (conventional, oxidation ditch, and sequence batch reactor [SBR]), and geographic locations (over a region of 68‐km radius) provided the other environmental variables. In addition, environmental variables covering *chemical stress, reactor scale, SRT, and reactor configuration* (fully aerobic vs. anoxic/aerobic) and *temporal drift* (interannual) were investigated in more details using samples collected during a pilot‐scale experiment at one of the treatment plants (LaPrairie‐WWTP) over a 2‐year period. For one of the pilot‐scale reactors, ozone was applied to the return activated sludge (RAS), which provided a chemical stress by enhancing bacterial mortality (decay) and modified the substrate composition in the system. In return, ozone effects on the RAS are likely to modify the AS community structure, a hypothesis that was tested by comparing the communities of two pilot‐scale reactors: RAS‐ozonated vs. nonozonated. The reactor‐scale gradient was studied at the same location by comparing pilot vs. full‐scale reactors. Interannual variations in the bacterial population assembly of full‐scale reactor were studied over a period of 4 years. The results generated from the combination of these studies enabled us to explore a range of environmental variables, which could potentially explain observed bacterial population assemblies and their response to abiotic changes.

## 2 Experimental Procedures

### 2.1 Sampling at full‐scale AS‐WWTPs

The mixed liquor samples were obtained at eight full‐scale AS‐WWTPs located within a 68‐km radius of the LaPrairie‐WWTP (Fig. S1). The selected plants differed in size, influent flow rates and characteristics, and treatment processes (Table S1). The sampling campaign was conducted over two time scales: samples were obtained during the same year in two seasons (summer and winter), and then one sample was obtained 4 years after to evaluate interannual stability in bacterial community composition. Samples were collected during August/September 2008 (summer of first year), February 2009 (winter of first year), February 2013 (winter 4 years after). In addition, three mixed liquor samples were obtained on consecutive weeks in August 2008, at one of the plants (Granby‐WWTP), to explore the population variation in weekly base. All mixed liquor samples, from each WWTP, after collection were transported to the laboratory on ice, and the solids were centrifuged and frozen at −80°C the same day until molecular biology analysis.

### 2.2 Pilot‐scale study with ozonation or return activated sludge

Ozonation of return activated sludge is a technique for minimization of waste biosolids production by chemical oxidation and enhanced solids degradation (Yasui & Shibata, 1994). Direct exposure of bacterial community to ozone has the potential to shape the community assemblies either through the high mortality induced by the ozonation or changes in the increased availability of organic substrates. Previously, we investigated the ozone impact on the microbial community composition by comparing two pilot‐scale reactors (a control and a RAS‐ozonated test reactor) in oxic condition (Isazadeh, Ozcer, & Frigon, 2014). Our comparison of FISH versus pyrosequencing experiment showed that some phyla were underrepresented in pyrosequencing method. Therefore, in this study, we used different forward primer sets (3 primers) and looked for more detailed operational conditions to explore the ozone effect on microbial population. Two experimental periods of 6–8 months were performed using pilot‐scale AS systems receiving the same wastewater as the LaPrairie‐WWTP; each periods were divided in phases of a few weeks with different AS operational conditions (SRT and conventional vs. anoxic/aerobic process) and ozone dosages to determine the potential of RAS‐ozonation effect on bacterial community (for additional details see supplementary materials and Table S1). Five and six biomass samples were collected, respectively, from the RAS‐ozonated and control (non RAS‐ozonated) reactor. Samples were collected at the end of each experimental phase (longer than 3 ×  SRT) to ensure the impact of the operation phase on the community composition. Solids from mixed liquor samples were centrifuged in the plant, and immediately frozen at −80°C until molecular biology analysis. Details of experimental design and operational conditions along with influent characteristics were presented in supplementary materials (Fig. S2 and Table S2) and elsewhere (Isazadeh, Feng, Urbina Rivas, & Frigon, 2014; Isazadeh, Urbana, Ozcer, & Frigon, 2015).

In parallel to the pilot‐scale reactor sampling, seven samples were also collected from the full‐scale LaPrairie AS‐WWTP to investigate the effect of scale (control pilot‐scale reactor samples vs. full‐scale reactor samples) and temporal variation on the bacterial community assembly.

### 2.3 Determination of microbial community composition

Genomic DNA was extracted from the mixed liquor suspended solids using a DNA extraction kit. Details of DNA extraction and PCR amplification are given in the supplementary material The V3‐V4 region of the 16S rRNA genes was then PCR amplified using a mixture of three forward primers and a reverse primer (to increase the phyla coverage), all with additional pyrosequencing adaptor sequences(Pinto & Raskin, 2012). The PCR amplicons were purified using a PCR purification kit; and sequenced using a GS FLX Titanium Sequencing machine (Roche Diagnostics, Hoffmann‐La Roche Ltd, Montreal, Canada). From all samples submitted for pyrosequencing, four samples of AS‐WWTPs (two sample of Vaudreuil‐WWTP and one sample of Salaberry‐WWTP) and one sample belong to control pilot‐scale study in the second phase of first year (Y1.PII) did not provide a reliable reads (less than 1000 read) and therefore has been removed from the downstream alpha and beta diversity analyses.

Postsequencing analysis was performed using the Qiime pipeline following the sequential analyses explained in the supplementary material.(Caporaso et al., 2010). We normalized the number of sequences prior to beta diversity to have the depth of coverage for even sampling.

### 2.4 Data analyses and variation partitioning of beta diversity

Detailed exploratory data analyses were carried out in the R software (V3.0.1) using the Vegan (V.2.08) (Oksanen et al., 2011) and cluster packages for heatmap (Maechler, Rousseeuw, Struyf, Hubert, & Hornik, 2011). To measure the statistical significance of within plant seasonal and interannual variations we used the multivariate dispersion approach based in distances to centroids (Anderson, Ellingsen, & McArdle, 2006). In this analysis *Betadisper* function in vegan was used for multivariate, permutation‐based hypothesis tests for differences in structure centroid and dispersion (beta diversity). Alpha diversities indices were compared based on a two‐sample *t* test using nonparametric method in Qiime. The details of this section are provided in the supplementary materials. Our initial study (Isazadeh, Ozcer et al., 2014) and other reports (Gobet, Quince, & Ramette, 2010) showed no significant changes in beta diversity results before and after denoising, therefore we did not used the denoised data. Principal coordinate analyses (PCoA) were performed based on weighted Unifrac and Hellinger distances (Legendre & Legendre, 2012). Since both distances resulted in similar ordination plots, the Hellinger distance is presented herein.

The relative importance of environmental variables shaping the community assembly was estimated using redundancy analysis (RDA) (Legendre & Legendre, 2012). In this part, analyses were performed based on community data transformed by the Hellinger distance. Influent characteristic data were log‐transformed and spatial distances measured with latitude‐longitude coordinates were converted into principal coordinates of neighbor matrices (PCNM) Eigen functions. The PCNMs were then used as explanatory variables to analyze the geographic location. The outcome of this approach relates to the fractions explained uniquely by each matrix and their combination. Unexplained fractions represented the parts which were not attributable to any of the applied explanatory matrix. For the eight full‐scale AS‐WWTPs, three explanatory matrices covering; influent characteristics, environmental, and spatial variations were used (Table S3, S4, and S5). The environmental explanatory matrix included the differences in processes (SRT, MLVSS, and HRT), seasons (winter vs. summer), and interannual (2008–2009 vs. 2013) variations. Partitioning of variations were performed using the *varpart* function of the Vegan Package (Oksanen et al., 2011). For LaPrairie‐WWTP, two explanatory matrices (containing either environmental or temporal explanatory variables) were used to investigate the variation in beta diversity (Table S3). The environmental matrix included reactors scale (control pilot‐scale vs. full‐scale), RAS‐ozonation (control vs. RAS‐ozonated pilot‐scale reactors), and season (summer vs. winter); whereas temporal matrix included the interannual variations (2008–2009 vs. 2013).

### 2.5 Sequence accession numbers

The 454 pyrosequencing reads have been deposited in the NCBI Sequence Read Archive, with the accession numbers: from SRS673933 to SRS673972.

## 3 Results

### 3.1 Bacterial community assemblies in regional full‐scale AS‐WWTPs

A total of 23 mixed liquor samples were acquired from eight full‐scale WWTPs. Mixed liquor samples were obtained in two seasons (winter and summer) during the first year and once after 4 years in winter season. This allowed the evaluation of seasonal and interannual differences in each treatment plant. From these samples, 83,248 of 16S rRNA gene amplicon sequence reads were obtained with the number of reads per sample ranging between 1,505 and 4,262 (average of 3,619 reads/sample). Unique OTUs were defined by grouping together sequence reads with ≥97% identities, which resulted in 5,610 OTUs. The average OTU richness within a sample was 643 with a range between 420 and 823 (Table 1).

**Table 1:** Sequence reads, number of OTUs (total and shared), and biodiversity numbers in eight AS‐WWTPs

AS‐WWTPsSampling period	Sequence reads and OTU richness				Simpson diversitynumber	Shannon diversity			
	No. of reads	OTUs	shared OTUs	% reads for shared OTUs		number	entropy (nat)	evenness	Top 3 abundant taxa^a^
Marieville									
2008 Summer^b^	3,591	561	140	47	36	123	4.81	0.22	*TM7‐1*,* Chloracidobacteria, Chitinophagaceae,*
2009 Winter^c^	3,617	834			167	376	5.93	0.45	
2013 Winter^c^	4,261	718			103	243	5.49	0.34	
Farnham									
2008 Summer	3,458	549	123	50	42	137	4.92	0.25	*Rhodobacter, Saprospiraceae, Chloracidobacteria*
2009 Winter	3,285	788			167	345	5.84	0.44	
2013 Winter	3,882	715			42	185	5.22	0.26	
LaPrairie									
2008 Summer	4,190	663	105	50	36	153	5.03	0.23	*OP11,TM7, Methylophilaceae,*
2009 Winter	3,400	656			39	155	5.04	0.24	
2013 Winter	3,311	717			16	98	4.58	0.14	
Cowansville									
2008 Summer	3,365	687	136	50	87	235	5.46	0.34	*Chloracidobacteria,, Acidobacteria, Caldilineaceae,*
2009 Winter	1,505	429			120	225	5.41	0.52	
2013 Winter	4,089	665			98	215	5.37	0.32	
Granby									
2008 Summer W1	3,215	704	187	47	82	238	5.47	0.34	*Caldilineaceae, Rhodobacteraceae, Rubrivivax,* d
2008 Summer W2	3,679	773			90	269	5.60	0.35	
2008 Summer W3	3,310	727			108	281	5.64	0.39	
2009 Winter	3,690	755			42	262	5.57	0.35	
2013 Winter	3,669	530			31	107	4.67	0.20	
Pincourt									
2008 Summer	3,699	488	80	35	15	77	4.34	0.16	*Flavobacteriaceae, Variovorax, Trichococcus*
2009 Winter	3,564	571			42	144	4.97	0.25	
2013 Winter	4,262	568			69	158	5.07	0.28	
Vaudreuil									*Flavobacterium, Arcobacter, Moraxellaceae*
2013 Winter	3,882	463			28	82	4.41	0.18	
Salaberry									
2008 Summer	4,249	641	185	50	27	134	4.90	0.21	*TM7*(*1* and *3*), *Flavobacteriaceae*
2013 Winter	4,075	618			69	171	5.14	0.28	

AS‐WWTPs, activated sludge wastewater treatment plants.

a Family level is reported unless the rank is not specified.

b August/September.

c February.

d Class *Burkholderiales*.

Comparison of the observed OTU alpha diversities (i.e., within sites) between samples gotten in summer 2008 and winter 2009 (only a few months apart) showed significantly higher diversity in the winter than in the summer (paired student *t* test, *p* < .05; Table 1). In general, this result is attributable to both higher OTU richness and evenness. The only noticeable counter example is the case of Cowansville WWTP where the community diversity was observed to be much lower in the winter than in the summer; however, this may be due to a lower number of sequence reads recovered for the Cowansville winter 2009 sample (Table 1). On average, the temperature drop between the summer and winter season, at these WWTPs, was about 8°C (averages for summer being 23°C and winter being 15°C).

**Figure 1:**
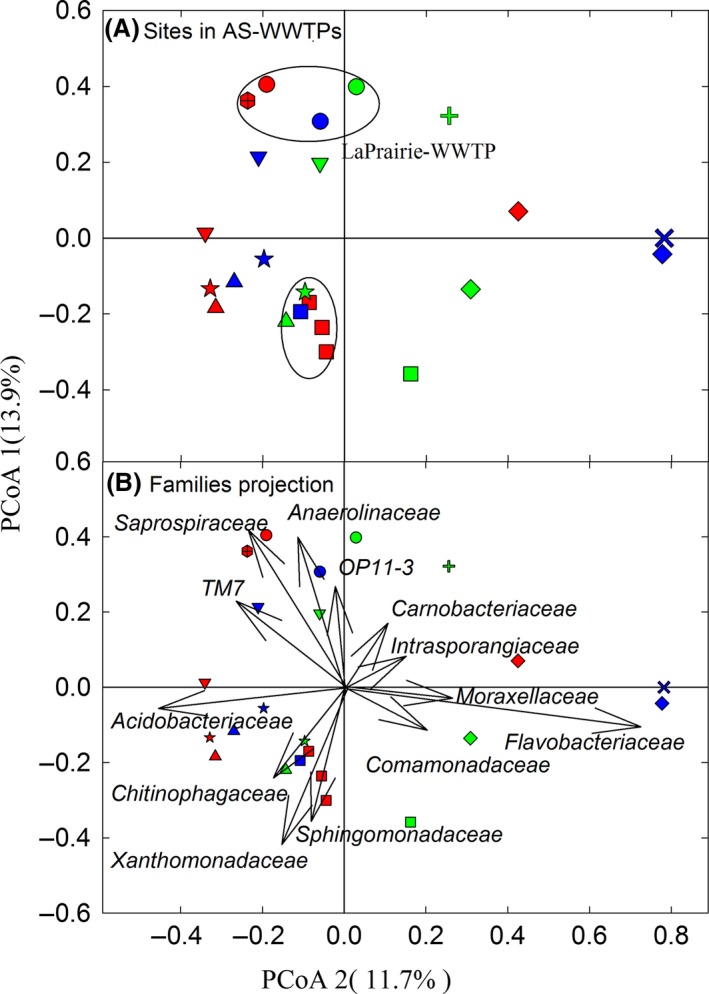
(A) PCoA plot representing Hellinger distances between community compositions for samples from eight activated sludge wastewater treatment plants (AS‐WWTPs) obtained in summer 2008 (red symbols), winter 2009 (blue symbols), and winter 2013 (green symbols). Each plant is indicated by a different symbol: Marieville (triangle‐down), Farnham (star), LaPrairie (circle), Cowansville (triangle‐up), Granby (rectangular), Pincourt (diamond), Vaudreuil (multiple), and Salaberry (plus) Circles in zone two and four highlight the location of samples taken from consecutive years and week, respectively. (B) Biplot of PCoA with projected score of major bacterial families which contributed to differences between the sites (see web for color version)

Differences in community compositions between samples obtained from the eight AS‐WWTPs were visualized using a PCoA of the Hellinger distances (Fig. 1A). In general, the differences in community composition between samples from different plants were relatively greater than the ones between samples of the same plant. This is true despite the fact that mixed liquor samples were taken in different seasons and 4 years apart for one set. Furthermore, the season did not seem to generate specific trends in the variations in the composition of the microbial community as the relative position of summer 2008 and winter 2009 samples varied between plants (Fig. 1A). This low impact of the season on the overall microbial community composition can clearly be seen in the samples from Granby. In this plant, the differences between the summer and winter samples were less than the differences observed between samples obtained on three consecutive weeks (Fig.1A). Altogether, these data suggested a relatively slow community turnover within plants and a relatively low impact of the season on the microbial community composition compared to differences between plants.

Although the relative differences in community composition between plants were much greater than ones within plants; it was still possible to identify a core set of phyla and families that were present in most samples. The average relative abundances of sequence reads for the main phyla in all plants were as follows: 36.1% for the *Proteobacteria* (with *Alpha‐, Beta‐, Gamma‐,* and *Delta‐proteobactria* classes accounting for 10.8%, 11.5%, 6.4%, and 2.4% of the reads, respectively), 27.6% for *Bacteroidetes* (with *Sphingobacteria*,* Flavobacteria,* and *Bacteroidia* accounting for 14.1%, 8.9%, and 1.8% of the reads, respectively) 9.0% for *Chloroflexi* (with the *Anaerolinea* class accounting for 8.4% of reads), 8.3% for *Acidobacteria*, 6.1% for *TM7*, 4.4% for *Actinobacteria*, and 3.7% for *Firmicutes* (Fig. S4a). Within these phyla, a group of common families could be defined as those present in at least six WWTPs based on the total number of reads that they represented (Fig. S6). Some of the most abundant of these families (and Table S6) were as follows: the *Flavobacteriaceae* and *Saprospiraceae* (phylum *Bacteroidetes*), the *Rhodobacteriaceae* and *Sphingomonadaceae* (class *Alphaproteobacteria*), and the *Comamonadaceae* (class *Betaproteobacteria*). It is mainly differences between the abundances of these families that is depicted in Figure 1.

Beside the core group of families, a group of plant‐specific and abundant (>6% reads from the plants) families could also be identified (Table S6). The main plant‐specific/abundant families observed were *Methylophilaceae* (at LaPrarie WWTP, class *Betaproteobacteria*)*, Bradyrhizobiaceae* (at Farnham WWTP, class *Alphaproteobacteria*)*, Thiotrichaceae* (at Granby WWTP, class *Gammaproteobacteria*), family *mb2424* from phylum *Acidobacteria* (at Farnham and Marieville WWTPs)*, Gordoniaceae* (at Pincourt WWTP, phylum *Actinobacteria*)*, Leptotrichiaceae* (at Vaudreuil; phylum *Fusobacteria*), *Gemmataceae* (at Cowansville WWTP, phylum *Planctomycetes*), and an unassigned family of the phylum *OP11* (at LaPrairie WWTP).

### 3.2 Bacterial community assembly in pilot‐scale experiments at LaPrairie‐WWTP

The pilot‐scale experiment conducted at the LaPrairie‐WWTP allowed us to investigate the impact of manipulated environmental variables on the microbial community treating real wastewater; specifically investigated were: scale of the reactors (pilot‐scale vs. full‐scale), SRT, reactor configuration (fully oxic vs. anoxic/aerobic) and chemical stress induced by ozonation of the RAS. Six samples were obtained from each of the pilot‐scale reactors (nonozonated control and RAS‐ozonated) at the end of each experimental phase (Table S2); in parallel, six samples were obtained from the full‐scale reactor. These 18 samples yielded a total of 57,316 for 16S rRNA sequence reads, with the numbers of reads per samples between 1,060 and 3,950 (3,371 on average, Table 2). Comparisons of the diversity measurements between the all reactors (i.e., among pilot‐scale reactors and between and pilot‐ and full‐scale reactors) did not reveal any significant difference (nonparametric *t* test on OTU Richeness, and Shannon and Simpson indices, *p* < .05; Table 2), suggesting that reactor's scale or the RAS‐ozonation process did not significantly affect the alpha diversity of the microbial communities in each reactor.

**Table 2:** Sequence reads, OTUs (total and shared), and biodiversity numbers for LaPrairie AS‐WWTP

LaPrairie‐WWTP	Sequence reads and OTU richness				Simpson diversitynumber	Shannon diversity		
	Reads	OTUs	Shared OTUs	% reads for shared OTUs		Number	Entropy(nat)	Evenness
Full‐scale reactor								
*1 years before pilot‐scale study*								
December	3,570	664	83	40	73	222	5.40	0.34
September	3,950	705			74	215	5.37	0.31
*Year 1*								
August	3,857	688			57	196	5.28	0.29
September	3,866	740			69	212	5.36	0.29
*Year 2*								
May	3,743	594			63	177	5.18	0.30
September	3,651	638			53	162	5.09	0.25
Pilot‐scale reactors								
*Control (non‐Ozonated)*								
***Year 1***								
Phase I—August	3,057	554	64	31	38	154	5.04	0.28
Phase II—September	N.A							
*Year 2*								
Start‐up‐May	3,408	659			106	253	5.54	0.38
Phase I—July	3,109	547			46	156	5.06	0.29
Phase II—September	3,714	753			88	262	5.57	0.35
Phase III—November	3,571	651			53	174	5.16	0.27
*RAS‐Ozonated*								
*Year 1*								
Phase I—August	3,485	673	42	24	102	240	5.48	0.36
Phase II—September	2,503	484			55	164	5.10	0.34
*Year 2*								
Start‐up‐May	3,878	689			110	255	5.54	0.37
Phase I—July	3,281	618			90	223	5.41	0.36
Phase II—September	1,060	330			89	175	5.17	0.53
Phase III—November	3,613	594			63	160	5.07	0.27

AS‐WWTPs, activated sludge wastewater treatment plants.

The average community composition comprised of eight phyla with relative abundances among the sequence reads higher than 1%. The most abundant phylum was *Proteobacteria* (38.5% of reads), with the classes *Alpha‐*,* Beta‐*,* Gamma‐*, and *Delta‐proteobacteria* accounting for 8.5%, 14.4%, 8.6%, and 6.6% of the reads, respectively. The phylum *Bacteroidetes* (19.8%) was mainly represented by the *Sphingobacteria* (15.0%) and *Flavobacteria* (3.9%) classes. *Chloroflexi* (12.9%) members almost entirely belonged to the class *Anaerolineae* (12.2%); while the phylum *Verrucomicrobia* (7.6%) was dominated by the genus *Prosthecobacter* (5.3%). Finally, the phyla *TM7* (3.7%), *Planctomycetes* (3.6%), *Acidobacteria* (2.3%), and *OP11* (1.3%) were also found among the most abundant ones (Fig. S4b). Similarities at the family level were also observed; most notably were the high abundance of two *Betaproteobacteria* families: the *Comamonadaceae* (6% of reads) and *Methylophilaceae,* (5%). These families were among the top 10 most abundant families in all mixed liquor samples (Table S7, Fig. S5).

**Figure 2:**
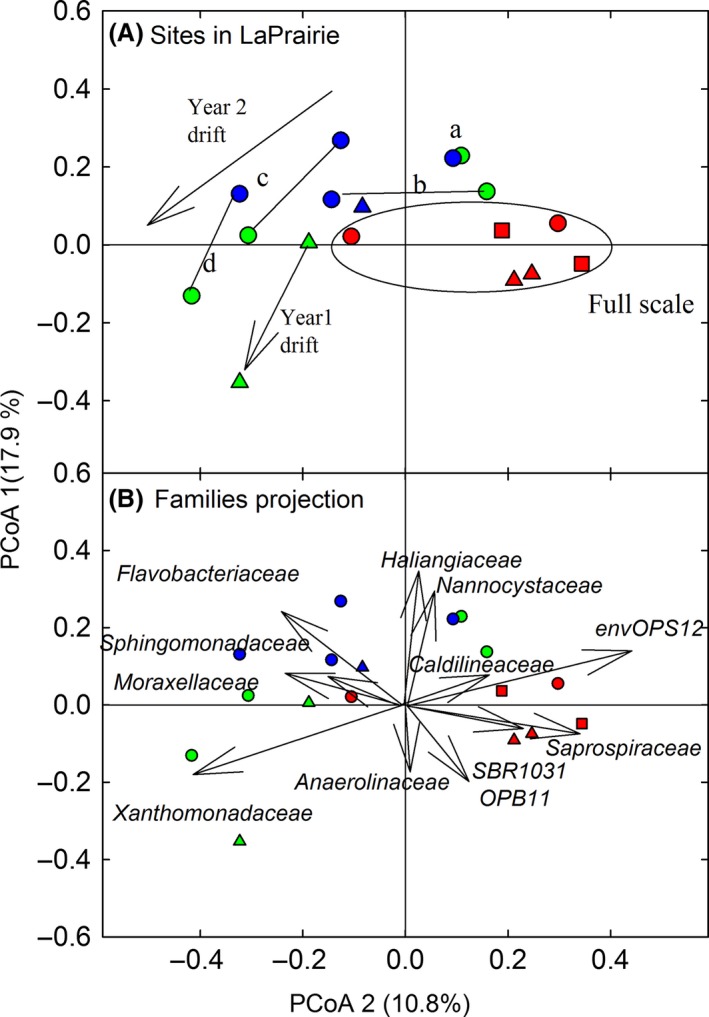
(A) PCoA of the Hellinger distances between community composition obtained by 16S rRNA gene amplicon sequencing of mixed liquor samples from the LaPrairie‐WWTP reactors: full‐scale [red symbols], control pilot‐scale [blue symbols], and return activated sludge (RAS)‐ozonated [green symbols]. Symbols represent sampling years: triangle for Year 1 and circle for Year 2 pilot‐scale experiment; square for sample before pilot‐scale experiment. Lines between symbols represents samples obtained at the same times from both pilot‐scale reactors and arrows indicate temporal drifts in community composition. For Year 2 pilot‐scale experiment, (Samples a) beginning of the experiment after 1‐month start‐up [May, same operations as Samples b but without ozonation], (Samples b) end of the anoxic/oxic with solids retention time (SRT) = 12 day phase [Phase I—July], (c) end of the fully aerobic with SRT = 12 day phase [Phase II—September], and (d) end of the fully aerobic with SRT = 6 day [Phase III—November]. (B) Biplot of PCoA with projected score of major bacterial families marking differences between samples (see web for color version)

Principal coordinate analysis (PCoA) was performed to visualize the differences in community composition (i.e., beta diversity). In the full‐scale reactor, the community composition did not vary much over the three sampling years as all the points appear close to each other on the PCoA plot (Fig. 2A). To provide an idea of magnitude of differences, the points reported for the LaPrairie‐WWTP in Fig. 1 (among WWTP differences) are equivalent to the points for the full‐scale reactor in Fig. 2; clearly, the variations in community composition during the pilot‐scale experiment remained smaller than the variations between full‐scale WWTPs. Nonetheless, specific changes in community composition could be observed in the pilot‐scale reactors during the experiment (Fig.1A); however, these changes did not appear related to the RAS‐ozonation process because the composition of both communities drifted in the same direction with the succession of operation phases (variation in SRTs and oxic vs. anoxic/aerobic configurations). Consequently, the similarity of the microbial composition between the two pilot‐scale reactors (i.e., the proximity of paired samples in Fig. 2A) remained relatively high throughout the study.

The main differences in microbial community compositions among the mixed liquor samples obtained during the pilot‐scale experiments are primarily due to a series of families from the class *Anaerolineae* (phylum *Chloroflexi*; families: *envOPS12*,* OP11*,* Caldilineaceae*,* Anaerolinaceae*, and *SR1*), and the family *Saprospiraceae* belonging to order *Sphingobacteriales* (phylum *Bacteroidetes*) (Fig. S5). These families explain for the most part the PCoA1 ordination (Fig. 2A). Together, the abundances of the class *Anaerolineae* population were higher in the full‐scale than in the pilot‐scale reactors; and they decreased in both pilot‐scale reactors (RAS‐ozonated and control) in Year 2 of the pilot‐scale experiment when the treatment systems were changed from anoxic/aerobic to completely aerobic. Reviewing the full‐scale WWTP operation data suggested that the front‐end of the aerobic plug‐flow reactor was deficient in aeration, and that significant denitrification occurred (data not shown). Therefore, the presence of *Anaerolineae* seemed to be related to the denitrification in this system. Thus variations in treatment process conditions and aeration efficiencies as opposed to specifically reactor scale may be the source of differences in community composition between the full‐scale and pilot‐scale reactors.

The family *Xanthomonadaceae* also change significantly in abundance in both pilot‐scale reactors through the Year 2 pilot‐scale experiment. When the SRTs decreased from ~12 day to ~6 day, *Xanthomonadaceae* increased in abundance; an observation similar to the one obtained during a previous pilot‐scale study at the same site during which the SRT was also 6 day (Isazadeh, Ozcer et al., 2014). Thus, low SRTs (i.e., high growth and dilution rates) appear to favor the growth of *Xanthomonadaceae* in this system.

### 3.3 Partitioning of the beta diversity

The first step in understanding the variations in community compositions is to correlate it with value of the state variables describing the conditions prevailing at the time of sampling (WWTP, influent composition, seasons, and process type). Such correlation would measure the explanatory power of each environmental variable. Initially, it may be more productive to measure the explanatory power of all available state variables together.

The quantification of the explanatory power of the available state variables was performed on the dataset of community compositions from the various full‐scale WWTPs. It was found that the influent characteristics could explain 21%, and the geographic locations could explain 10–25% of the variance in community compositions (Table 3). However, most of the explanatory power of these two sets of state variables was shared. The environmental conditions (process types and seasons) could only add 5% to the explanation of the variance in community compositions (Table 3); thus, most of the composition variance among plants remained unexplained (74%). This data suggest that the influent characteristics and/or the geographic location are important explanatory variables. Yet, the high unexplained variance suggests that the explanatory variables used or the 16S rRNA community composition contain only weak ecological signals.

**Table 3:** Variation partitioning results

Sampling Sites	Explanatory factors	Variance fractions	
		Explained	Unexplained
8 AS‐WWTPs			
	Influent a	0.21	0.79
	[a] Influent + [b] Environmental^b^	0.21 + 0.00 + 0.05^f^	0.74
	Geographic locations^c^	0.10	0.90
	[a] Geographic locations + [b] Influent	0.04 + 0.21 + 0.00	0.75
Granby‐WWTP			
	[a] Season + [b] Interannual	0.00 + 0.05 + 0.21	0.74
LaPrairie‐WWTP			
	Environmental d	0.11	0.89
	Interannual e	0.07	0.93
	[a] Environmental + [b] Temporal	0.06 + 0.05 + 0.02	0.87

Details of the explanatory factors

a Influent = Industrial fraction (%) + flow rate + COD + BOD_5_ + VSS concentrations.

b Environmental = process types (sequence batch reactor [SBR] vs. conventional AS vs. Oxidation ditch) and season (winter vs. summer).

c Geographic locations defined by principal coordinates of neighbor matrices (PCNM) eigenfunctions.

d Environmental = scale (full‐scale vs. pilot‐scale) + treatment (fully aerobic, anoxic/aerobic, SRTs, RAS‐ozonated) + season (winter vs. summer).

e Interannual (pilot‐scale study Year 1, pilot‐scale study Year 2, Year before pilot‐scale study).

Interpretation of the combined explained variance fractions

f explained fractions are: [explained solely by a] + [shared explanation (a∩b)] + [explained solely by b].

The composition variance partitioning was also performed with the Granby WWTP samples. At this plant, seasonal variations showed no contribution and interannual variations contributed to 21% of the observed variations, respectively, with an additional 5% of the variance explanation shared by the two sets of variables (Table 3). Along with the complete full‐scale dataset, this result suggests that a strong underlying drift in community composition is more important in determining the composition than the summer/winter seasonal effect.

The variance among the LaPrairie‐WWTP community compositions (full‐scale and both pilot‐scale reactors) was partitioned over a set of variables describing the environmental conditions (namely scale of the reactor, process operation, and season), and another one describing the sampling year. Together, these two sets of state variables could only explain 13% of the variance in OTU community composition (Table 3).

## 4 Discussion

### 4.1 Core bacterial community of conventional activated sludge systems

The OTU structures of the AS‐WWTP bacterial communities determined in the current investigation were in line with previously observed studies; the OTU distributions followed a strong power law rank‐abundance model in which the top 10–20 OTUs (grouped in as many genera) accounting for 70–80% of the sequence reads, and a long tail of OTUs (~150 genera) accounting for the remaining reads (Hoffmann et al., 2007; Xia et al., 2010). The identities and abundances of observed phyla were also highly similar to the previous studies (Seviour & Nielsen, 2010; Zhang et al., 2012).

The resemblance of the community assemblies observed in this study were high over time and between WWTPs as determined at the levels of OTU (97%‐identity cut‐off), family and phylum (Table S6). Such similarities in bacterial community have been reported by others when comparing bacterial community assemblies at AS‐WWTPs in China and in North America (Zhang et al., 2012). In this study, common families (Table S6) were identified as forming the core of the conventional AS microbial communities. Given the relatively few high abundant taxa, it appears that the first task on which environmental microbiologists and engineers should focus is to explain the mechanisms leading to the formation of this core group of families within the AS community. It is possible that the presence of such core families is the results of similar compositions of municipal wastewaters; which are relatively constant in spite of differences in human municipal sources (rural vs. urban), income levels, and food cultural habits (Tchobanoglous, Burton, Stensel, H. D., 2003). In addition, the AS microbial communities are subjected to seeding by the human gut microbiome released into the sewer systems, which can also influences the community assemblies (Curtis, Wallbridge, & Sloan, 2009; Shanks et al., 2013). Describing the mechanisms explaining the presence of core microbial populations is at the top most agenda items for the upcoming years in wastewater microbiology.

The differences in microbial communities revealed by the principal coordinate analysis were mainly related to variations in the abundance of these core microbial families (Fig. 1). Among the families describing the differences between plants are the ones belonging to the class *Anaerolineae* and the family *Saprospiraceae* (phylum *Bacteroidetes*), which are pointing upward along PCoA2, and the family *Xanthomonadaceae*, which is pointing downward along PCoA2 (Fig. 1). This pattern reveals a positive correlation in the abundances of the class *Anaerolineae* and the family *Saprospiraceae*, and a negative correlation with the abundances of the family *Xanthomonadaceae*. The same correlations were present within the data of the pilot‐scale reactors At LaPrairie WWTP despite a much reduced diversity in operation conditions. Finally, the same correlations were also observed in other studies studying either differences between plants or temporal variations within a plant (Ju & Zhang, 2015; Vuono et al., 2015). Since the variations in operation conditions were very different in all these datasets, this suggests some biotic interactions (e.g., competition or cooperation) exist between the members of these families. While members of the family *Saprospiraceae* have been identified as strong protein‐hydrolysers (Xia, Kong, & Nielsen, 2007), the role of members of the two other taxa (class *Anaerolineae* and family *Xanthomonadaceae*) remain to be defined.

### 4.2 Plant‐specific abundant families of conventional activated sludge systems

Despite the presence of a core set of families, this study identified plant‐specific abundant families; their presence in some cases can be directly linked to environmental variables. The high prevalence of *Methylophilaceae* (main genus: *Methylotenera*) in all the LaPrairie‐WWTP samples (full and pilot‐scale reactors) highlighted the importance of influent wastewater composition on shaping the bacterial community structure. Analysis of the influent wastewater from LaPrairie‐WWTP revealed a high methanol and nitrate concentrations, which in turn explains the high abundance of the *Methylotenera* genus in this AS‐WWTP (Isazadeh, Ozcer et al., 2014).

At other full‐scale AS‐WWTPs studied, a number of plant‐specific abundant bacterial families were identified (Table S6). Among these, the genera from the families *Gordoniaceae*,* Thiotrichaceae* are well‐known for their implication in solid–liquid separation problems including bulking and foaming due to their filamentous morphology (Martins et al., 2004). The presence of these organisms has also been linked to influent compositions. For examples, members of the *Gordoniaceae* are present at high lipid loading rates (Frigon, Muyzer, van Loosdrecht, & Raskin, 2006); and members of the *Thiotrichaceae* are present at high volatile fatty acid concentration and in the presence of sulfides as they are capable of mixotrophic growth (Martins et al., 2004). Although the links between the influent composition and other environmental factors is not established for all the plant‐specific highly abundant populations, these observations reported here demand further investigations of their role in activated sludge microbial communities.

### 4.3 Level of explanation of variation in microbial community composition

Partitioning the variance in community compositions among AS‐WWTP revealed that influent compositions and geographic locations influence most the bacterial community structures among the factors tested, although these variables did not explain more than 26% of the observed variations. It was in general difficult to differentiate the contributions of influent compositions and geographic locations on the bacterial community structures. This may have been in part due to the lack of chemical descriptors of the influent composition, and further study will be required to properly establish the relative importance of each factors.

Geographic locations of WWTPs can affect bacterial population assemblies either by local weather effects (e.g., rainfall), or by watershed effects such as soil composition, which may influence the seeding of treatment plants. However, the proximity of the AS‐WWTPs in this study (136 km between the most distant plants) does not seem to argue for local weather effects. Interestingly, the Pincourt and Vaudreuil WWTPs (appearing on the right of the PCoA plot, Fig. 2) are both on the north shore of the St‐Laurent's River. Also, the Cowansville, Farnham and Grandby WWTPs (appearing in the bottom left quadrant of the PCoA plot, Fig. 2) are all located in the Yamaska River watershed. Therefore, it is possible that watersheds influence the community structure at WWTPs.

In spite of the observed importance of influent wastewater characteristics on community composition, traditional characterization of municipal wastewater is likely insufficient to understand WWTP communities. For example, the prevalence of *Methylophilaceae* in the case of LaPrairie‐WWTP was linked to the presence of methanol and nitrate in the influent (Isazadeh, Ozcer et al., 2014). Consequently, wastewater treatment microbial ecologists need to go beyond the conventional influent characterization such as BOD_5_, COD, total phosphorus and ortho‐phosphate, total Kjeldhal nitrogen, and volatile suspended solids to describe the composition of incoming wastewater with a high enough precision to understand the community structure and meaningfully explore the structure‐functions relationships of heterotrophs. Domestic wastewater contains: proteins (40%–60%), carbohydrates (25%–50%), fats and oils (10%), urea and a large number of organic compounds including pesticides and herbicides (Bitton, 2011). Additionally, influence of local industrial wastewater in contributing specific organics should not be neglected. Therefore, wastewater characterization should reveal the diverse organic content of wastewater. Such characterization would help linking the community assembly and wastewater composition.

Even though changes in some populations were linked to variations in environmental variables, the observed variance in obtained community data with high‐resolution 16S rRNA gene amplicon pyrosequencing could not be explained at more than 13% for the LaPrairie‐WWTP full‐scale and pilot‐scale reactors. Thus, the major fraction of the community assembly variance remains unexplained, suggesting that the hypothesized variables namely *continuous chemical stress, reactor scale, and SRT* were minor factors in explaining the community structures. This could be surprising in the case of RAS‐ozonation (*continuous chemical stress*), which effectively inactivates approximately 18% of the microbial biomass every day (Isazadeh, Feng et al., 2014). Nonetheless, the difficulty of establishing clear relationships between the community composition dynamics and variation in operational factors was also observed by other authors. For example, Saikaly, Stroot, and Oerther (2005) and Akarsubasi et al. (2009) observed a slight correlation between changes in SRT and bacterial population assemblies, but had difficulty to show statistically meaningful links between them. Consequently, both groups, who worked with rRNA‐gene‐targeted TRFLP or DGGE, concluded that higher resolution molecular biology techniques (i.e., deeper sampling of community diversity) would reveal the link between the community composition and operational changes. This study was performed by pyrosequencing and it reached the same results. Therefore, it did not support such speculation.

This being said, the results of community composition variance partitioning could be related to other mechanistic and theoretical reasons, namely that communities could be assembled by neutral mechanisms (Hubbell, 2001). Curtis and Sloan (2006) used the stochastic approach based on random‐assembly to describe autotrophic populations of ammonia oxidizing bacteria (AOB) in wastewater systems. Ofiţeru et al. (2010) suggested that neutral community models should form the foundation of any description of open biological system. There are also room for a spectrum of theoretical mechanisms between niche‐assembly and random‐assembly approach if one considers that the niche exclusion principle does not absolutely limit community diversity and that even competition may be compatible with more neutral community assembly models. For example, competition dynamics can generate periodic or chaotic oscillations in the abundances of species that can generate niches with more species than limiting resources (Huisman & Weissing, 1999). This last finding was also supported by a modeling approach based on game theory which showed that the diversity in a local niche is more dependent on the meta‐community diversity for a given function than the number of limiting resources associated with the niche (Allesina & Levine, 2011). These considerations suggest that much more data (i.e., limited number of samples in this experimental design along with missing data) and theoretical development will be necessary to understand community assemblies in biological wastewater treatment systems.

Finally, this study provides important validations of pilot‐scale studies in understanding the microbial community dynamics. In this study, the community compositions between the full‐scale and pilot‐scale reactors at LaPrairie‐WWTP were highly similar compared to variations between plants (Fig. 1), and the diversities was essentially the same at both scales (Table 2) despite a difference of 16,000 ×  in the size of the bioreactors. The lack of implication of the physical scale of treatment plants in significantly structuring the community in the reactors at the LaPrairie‐WWTPs has two important implications. First, pilot‐scale studies can faithfully represent full‐scale treatment plants. Second, it argues against the principle that larger bioreactors are necessary to ensure efficient and stable microbial communities (Curtis, Head, & Graham, 2003; Valentín‐Vargas et al., 2012). Consequently, it appears that the size of treatment plant infrastructures remains an economic decision, and it is not a population stability issue.

## Funding Information

This study was funded by an NSERC Collaborative Research and Development Grant (CRDPJ 417654 – 11) in partnership with Air Liquide Canada and Régie d'Assainissement des Eaux du Bassin LaPrairie.

## Conflict of Interest

The authors declare no conflict of interest.

## Supporting information


**Figure S1.** Location of eight AS‐WWTPs used in this study. South shore of Montreal, Canada
**Figure S2.** Configuration of pilot‐scale reactors. Dashed lines in the aeration tanks depict the perforated Plexiglas wall used to separate anoxic and oxic chambers in the Year 2 of experiment.
**Figure S3.** Rarefaction curve; a) full‐scale WWTPs, and (b) LaPrairie‐WWTP
**Figure S4.** Abundancy of sequence read in Phylum/Class level at: (a) full scale WWTPs, and (b) LaPrairie‐WWTP**.** Note that two most abundant phylum (i.e., *Proteobacteria* and *Bacteroidetes*) are presented in class level. In panel b, Y0, Y1, and Y2 represent 1 year before pilot‐scale study, first, and second year of pilot‐scale, respectively.
**Figure S5**. Heat map of sites and of top 10 highly abundant families observed in LaPrairie‐WWTP reactors. For the sample name, F, O3, and C represent; Full‐scale, RAS‐ozonated, and Control reactor, respectively, and Y.0, Y.1, and Y.2 show the sampling time a year before and the first and second year of pilot‐scale study, respectively.
**Figure S6.** Abundance of shared sequences in WWTPs.
**Table S1.** Characteristic of AS‐WWTPs (full ‐scale).
**Table. S2.** Summary of pilot‐scale reactors operation and experimental phases over 2 years.
**Table S3.** Environmental explanatory matrix for the eight AS‐WWTPs.
**Table S4.** Environmental explanatory matrix for Granby‐WWTP.
**Table S5.** Environmental explanatory matrix for LaPrairie AS‐WWTP.
**Table S6.** Core and rare bacterial population observed in full‐scale AS‐WWTPs in family level.
**Table S7**. Observed abundant families in LaPrairie‐WWTP reactors.
