# Supplementary material for: Bacterial community assembly in activated sludge: mapping beta diversity across environmental variables
Source: Microbiologyopen. 2016 Oct 19;5(6):1050–60. doi: 10.1002/mbo3.388 (PMC5221439; doi:10.1002/mbo3.388)
Supplement: Supplementary file 1 — Figure S1. Location of eight AS‐WWTPs used in this study. South shore of Montreal, Canada Figure S2. Configuration of pilot‐scale reactors. Dashed lines in the aeration tanks depict the perforated Plexiglas wall used to separate anoxic and oxic chambers in the Year 2 of experiment. Figure S3. Rarefaction curve; a) full‐scale WWTPs, and (b) LaPrairie‐WWTP Figure S4. Abundancy of sequence read in Phylum/Class level at: (a) full scale WWTPs, and (b) LaPrairie‐WWTP. Note that two most abundant phylum (i.e., Proteobacteria and Bacteroidetes) are presented in class level. In panel b, Y0, Y1, and Y2 represent 1 year before pilot‐scale study, first, and second year of pilot‐scale, respectively. Figure S5. Heat map of sites and of top 10 highly abundant families observed in LaPrairie‐WWTP reactors. For the sample name, F, O3, and C represent; Full‐scale, RAS‐ozonated, and Control reactor, respectively, and Y.0, Y.1, and Y.2 show the sampling time a year before and the first and second year of pilot‐scale study, respectively. Figure S6. Abundance of shared sequences in WWTPs. Table S1. Characteristic of AS‐WWTPs (full ‐scale). Table. S2. Summary of pilot‐scale reactors operation and experimental phases over 2 years. Table S3. Environmental explanatory matrix for the eight AS‐WWTPs. Table S4. Environmental explanatory matrix for Granby‐WWTP. Table S5. Environmental explanatory matrix for LaPrairie AS‐WWTP. Table S6. Core and rare bacterial population observed in full‐scale AS‐WWTPs in family level. Table S7. Observed abundant families in LaPrairie‐WWTP reactors. [file MBO3-5-1050-s001.pdf]

Bacterial community assembly in activated sludge: mapping beta diversity across environmental gradients

Siavash Isazadeh, Shameem Jauffur, and Dominic Frigon*

Department of Civil Engineering and Applied Mechanics, McGill University, 817 Sherbrooke Street West, Montreal, QC, H3A 0C3, Canada

*Corresponding author

Email: dominic.frigon@mcgill.ca

Tel: +1-514-398-2475

FAX: +1-514-398-7361

1. Materials and Methods

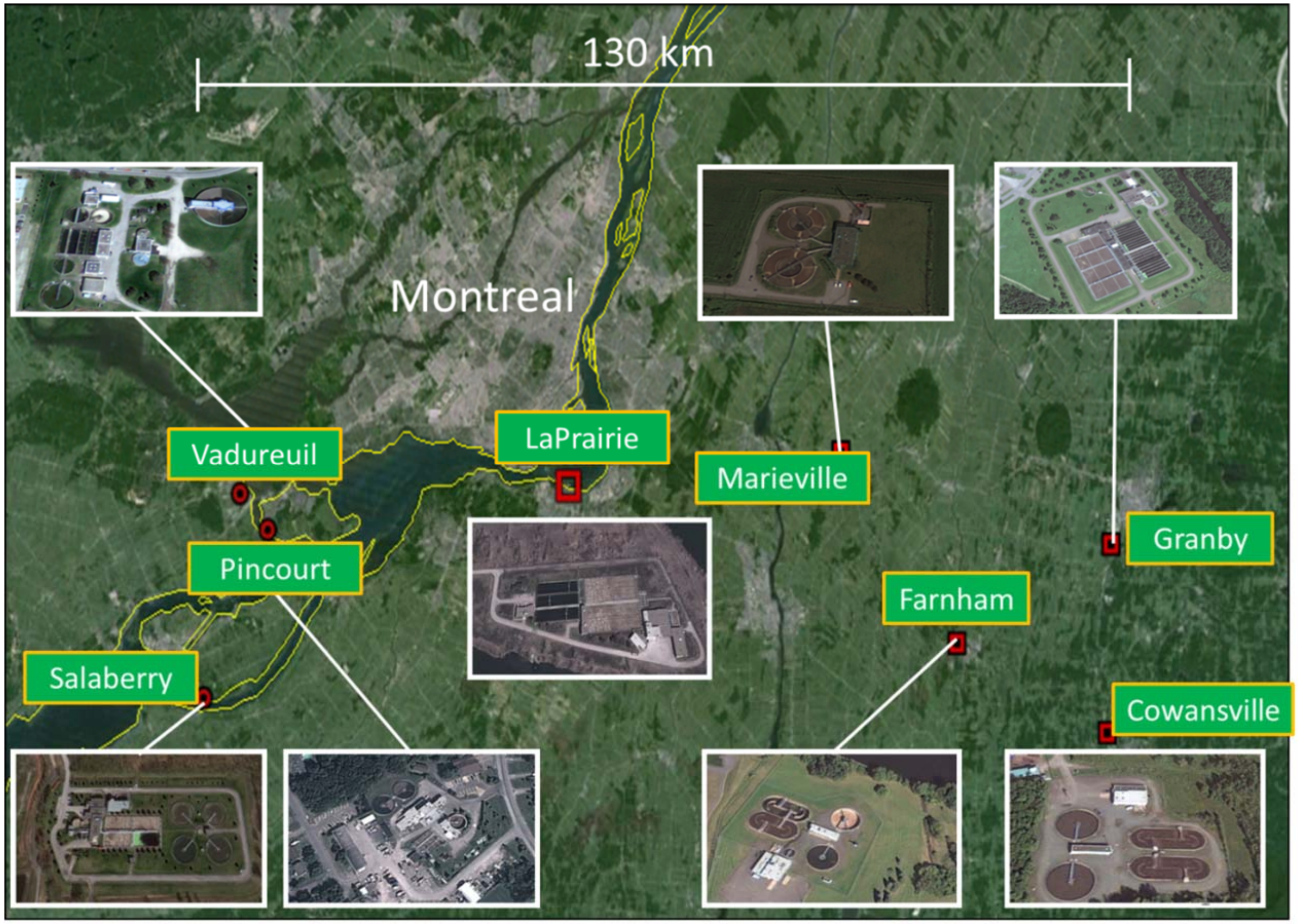

Fig. S1. Location of 8 AS-WWTPs used in this study. South shore of Montreal, Canada

Table S1. Characteristic of AS-WWTPs (full -scale).

AS-WWTPs	Process [a]	# of samples	Operational Parameters				Influent Characteristics			Geographic Position	
			Flow rate (m^3/day)	SRT (day)	HRT (h)	MLVSS (mg/L)	Influent Composition[b] (%)	COD (mg/L)	BOD_5 (mg/L)	Latitude N	Longitude W
Marieville	OD	3	5,000	25	12	3200	80+20	250	128.7	45°26'20.28"	73° 9'51.40"
Farnham	OD	3	6,000	80	48	6080	80+20	206	30	45°17'21.90"	72°59'35.05"
LaPrairie	CA	3	65,000	8	12	1850	45+55	333	143	45°24'16.48"	73°33'22.06"
Cowansville	OD	3	14,000	10	18	4910	90+10	233	46	45°13'16.55"	72°46'30.41"
Granby	CA	4	55,000	7	20	3116	50+50	468	231	45°22'17.45"	72°46'23.98"
Vaudreuil	SBR	1	18,000	5	3	3000	50+50	285	120	45°23'25.30"	74° 1'37.34"
Pincourt	CA	3	6,000	15	8	2121	90+10	316	102	45°23'25.30"	74° 1'37.34"
Salaberry	CA	2	57,000	25	12	2500	27+6(+57)	245	95	45°13'34.61"	74° 4'20.44"

a: OD;Oxidation Ditch CA; Conventional Aeration, SBR; Sequence Bach Reactor, Carrousel is a process based on the principals of oxidation ditch

b: Influent Composition in (%) Residential + Industrial (+Infiltration)

1.1 Pilot-scale reactor

Pilot scale reactors comprise of control and a RAS-ozonated test reactor, each with a total volume of 1.7 m^3 including the secondary clarifier. The hydraulic residence time (HRT) in the both reactors was 12 h. The reactors received the same municipal wastewater as the full-scale plant. The operational conditions and ozone dosages were varied in order to assess the potential of RAS-ozonation to reduce the excess biosolids production. In Year 1, both pilot-scale reactors were operated under fully aerobic conditions with a target SRT of around 6 days for the non-ozonated control reactor. The ozone dosage varied from medium (5.9±0.4 mg-O_3/[g-$VSS_{inventory}$.d]) in Phase I to high (10.3±0.7 mg-O_3/[g-$VSS_{inventory}$.d]) in Phase 2. In Year 2, the ozone dosage was kept constant (average of 9.2±0.2 mg-O_3/[g-$VSS_{inventory}$.d]) throughout the experiment and the operational conditions were varied through three experimental phases. In Phase 1 with a SRT of ~12 d, the reactor was split into an anoxic section and an aerobic compartment with a recirculation rates equaled to ~4 times of the influent flow between the section which provided for denitrification/nitrification activities to occur in the reactor. In Phase 2, the two reactor sections were operated under aerobic conditions with a SRT of ~12 d. Finally, in Phase 3, aerobic conditions were maintained, but the SRT was reduced to ~6 d. Each phase lasted for a minimum of 3 SRTs to reach steady-state conditions (Table S1).

Table. S2. Summary of pilot-scale reactors operation and experimental phases over 2 years.

Study Year<br>Operation Phase	Length (days)	Operation[b]	Control Reactor Target SRT (days)	Ozone Dose (mg-O3/g-VSS.d^{-1})	Reduction in Biosolids Production (%)
Year 1: Single treatment operation and high ozone dose					
Start-up	47		6	0	NA
Phase 1*	40	Aerobic	6	5.9±0.4	13±1
Phase 2*	34		6	10.3±0.7	53±6
Year 2: Variable operation and a single ozone dose					
Start-up*	60	A/O	12	0	NA
Phase 1*	100	A/O	12	7.3±0.2	22±2
Phase 2*	36	Aerobic	12	8.9±0.1	19±2
Phase 3*	40	Aerobic	6	11.4±0.2	18±2 [c]

a: NA: Not applicable

b: O:Fully Aerobic and A/O: Anoxic/ aerobic

c: The recirculation pump of the RAS-ozone contactor caused a decrease in the COD solubilization efficiency between Years 1 (5.26 g-COD/g-O_3) and Year 2 (2.13 g-COD/g-O_3).

* : Symbol represents the phases in which biomass sample were taken for the community analysis at the end of phas

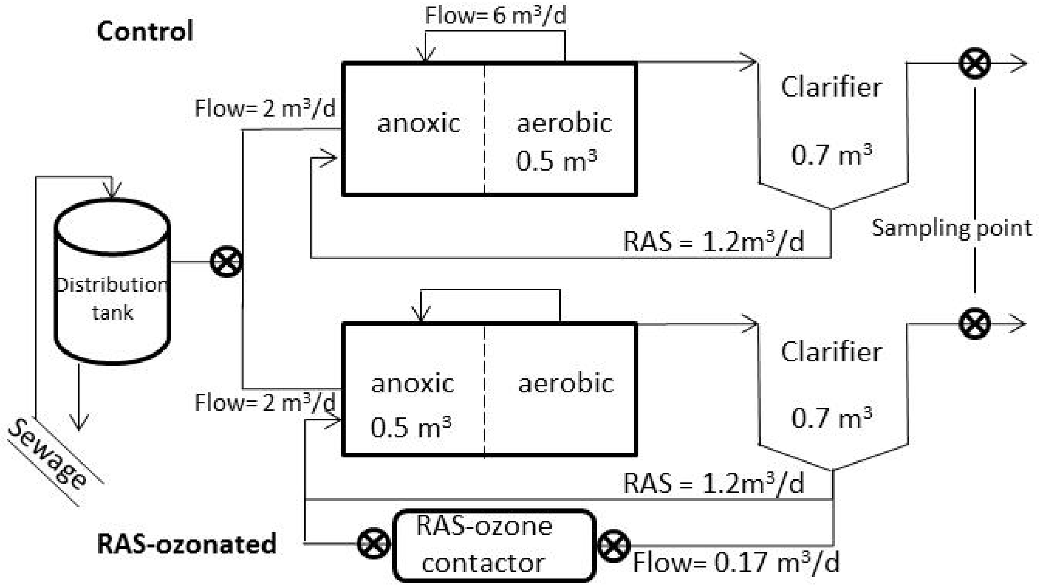

Fig. S2. Configuration of pilot-scale reactors. Dashed lines in the aeration tanks depict the perforated Plexiglas wall used to separate anoxic and oxic chambers in the Year 2 of experiment.

1.2 DNA extraction and sequencing

Genomic DNA was extracted from the mixed liquor samples using DNA extraction kit (*MoBio* UltraClean^{TM} Fecal DNA Kit, Mo Bio Laboratories Inc., Carlsbad, CA, USA). The DNA was amplified by PCR using a mixture of 3 forward primers (5'-CCTACGGGRGGCAGCAG-3', 5'-ACWYCTACGGRWGGCTGC-3' and 5'-CACCTACGGGTGGCAGC-3') targeting the hypervariable V3 region (*E.coli* position: 338) and 1 reverse primer (5'-TACNVGGGTHTCTAATCC-3') targeting the hypervariable region V4 (*E.coli* position:802)(Pinto and Raskin 2012). The primers sequences were tagged with pyrosequencing emulsion PCR adaptors (24 bp) and the reverse primer contained a specific barcode (14 bp). The PCR thermocycling conditions were as follows: 94 °C for 5 min, 30 cycles of 94°C for 1 min, 55 °C for 30 s, 72 °C for 1.5 min followed by a final extended elongation at 72°C for 8.5 min. The PCR amplicons were purified using PCR purification kit (*MoBio* UltraClean PCR Clean-UP Kit, Mo Bio Laboratories Inc., Carlsbad, CA, USA). The amplicon concentration of each sample was determined using the Quant-iT™ PicoGreen kit (Invitrogen, USA) and normalized to a concentration of 50 ng/µl. The PCR products were then pooled and their quality assessed by the Bioanalyzer 2100 (Agilent Technologies, Montreal, Quebec, Canada) to ensure the purity of the amplicons. Purified amplicons were subjected to emulsion PCR, based on the Roche-454 Life Science Protocol and eventually sequenced by the GS FLX Titanium Sequencing machine (Roche Diagnostics, Hoffmann-La Roche Ltd, Montreal, Canada).

1.3 Sequence processing and statistical analysis

Sequences reads first trimmed for barcodes and primers, and the ones with quality scores lower than 25 and length shorter than 200 bp were excluded from downstream analyses. Sequences

were clustered at 97% sequence similarity with Uclust (Edgar 2010) and taxonomic assignment was performed using the RDP classifier (Wang et al. 2007). Sequence reads were aligned to the Greengenes core reference alignment (DeSantis et al. 2006) using PyNAST (Caporaso et al. 2010) to capture the beta diversity based on phylogenetic distances in Unifrac (Lozupone and Knight 2005). A number of other descriptive and statistical methods inside the Qiime pipeline including, alpha diversity, diversity indices, and core microbial analysis were applied to acquire better understanding of the bacterial community structure and dynamics.

Table S3. Environmental explanatory matrix for the 8 AS-WWTPs.

	Process [a]		Season [b]	Temporal [c]
	SBR[d]	Oxidation Ditch		
Marieville				
2008 Summer	0	1	1	0
2009 Winter	0	1	0	0
2013 Winter	0	1	0	1
Farnham				
2008 Summer	0	1	1	0
2009 Winter	0	1	0	0
2013 Winter	0	1	0	1
LaPrairie				
2008 Summer	0	0	1	0
2009 Winter	0	0	0	0
2013 Winter	0	0	0	1
Cowansville				
2008 Summer	0	1	1	0
2009 Winter	0	1	0	0
2013 Winter	0	1	0	1
Granby				
2008 Summer	0	0	1	0
2009 Winter	0	0	0	0
2013 Winter	0	0	0	1
Pincourt				
2008 Summer	0	0	1	0
2009 Winter	0	0	0	0
2013 Winter	0	0	0	1
Vaudreuil				
2013 Winter	1	0	1	1
Salaberry				
2008 Summer	0	0	1	0
2013 Winter	0	0	0	1

a: conventional activated sludge is represented by SBR=0 and Oxidation ditch=0
b: winter= 0 , summer=1
c: 2008-2009=0, 2013=1
d: SBR: Sequencing batch reactor

Table S4. Environmental explanatory matrix in Granby-WWTP.

Sample	Year	Season
2008 Summer W1	0	1
2008 Summer W2	0	1
2008 Summer W3	0	1
2009 Winter W3	0	0
2013 Winter	1	0

Table S5. Environmental explanatory matrix in for LaPrairie AS-WWTP.

LaPrairie-WWTP	Scale [a]	Treatment [b]	Season [c]
**Full-scale**			
1 years before pilot-scale study			
December	0	1	0
September	0	1	1
Year 1			
August	0	1	1
September	0	1	1
Year 2			
May	0	1	0
September	0	1	1
**Pilot-scale reactors**			
Control (non ozonated)			
Year 1			
Phase I-August	1	1	1
Phase II -September	N.A	N.A	N.A
Year 2			
Start-up-May	1	1	0
Phase I-July	1	1	1
Phase II-September	1	1	1
Phase III-November	1	1	0
RAS-ozonated			
Year 1			
Phase I-August	1	0	1
Phase II -September	1	0	1
Year 2			
Start-up-May	1	0	0
Phase I-July	1	0	1
Phase II-September	1	0	1
Phase III-November	1	0	0

a: pilot-scale=0, full-scale=1

b: no ozone exposure =1, ozone exposure = 0

c: winter=0, summer=1

2. Results and discussion

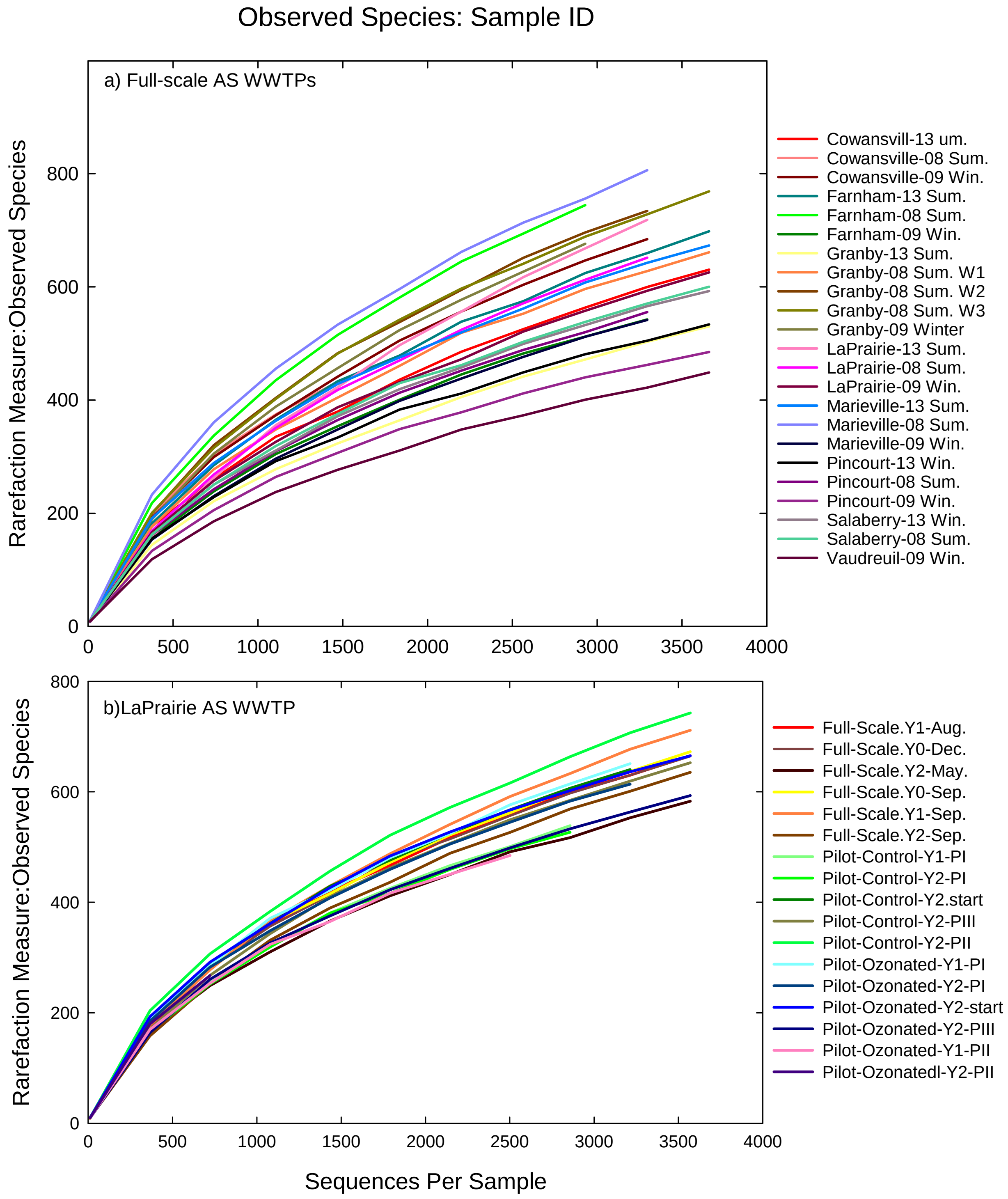

Fig.S3 Rarefaction curve; a) full scale WWTPs, and (b) LaPrairie-WWTP

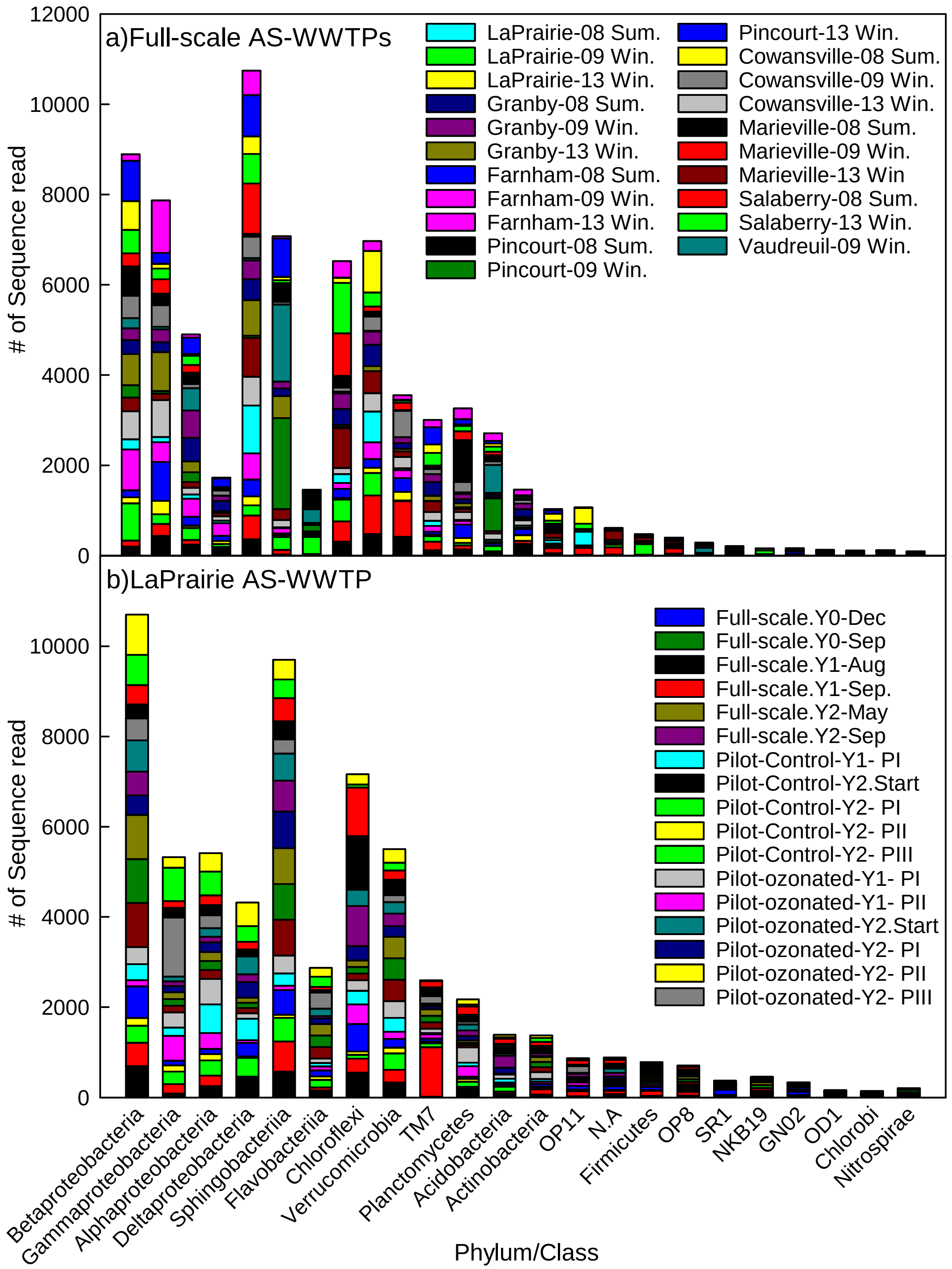

Fig. S4 Abundancy of sequence read in Phylum/ Class level at: (a) full scale WWTPs, and (b) LaPrairie-WWTP**.** Not that two most abundant phylum (i.e., *Proteobacteria* and *Bacteroidetes*) are presented in class level. In panel b, Y0, Y1, and Y2 represent 1 years before pilot-scale study, First and second year of pilot-scale, respectively.

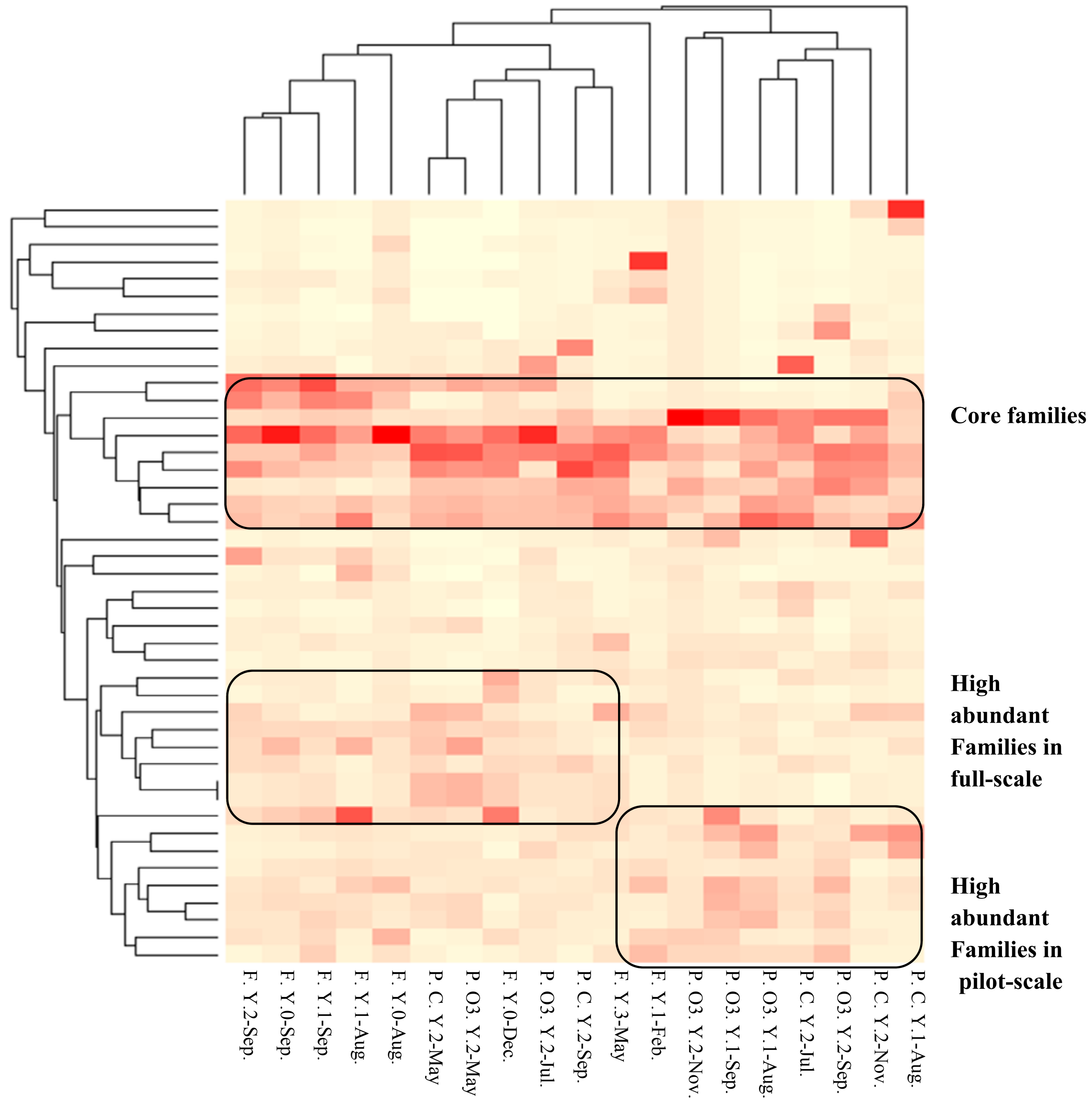

Fig. S5. Heat map of sites and of top 10 highly abundant families observed in LaPrairie-WWTP reactors. For the sample name, F, O3 and C represent; Full-scale, RAS-ozonated, and control reactor, respectively, and Y.0, Y.1 and Y.2 show the sampling time a year before and the first and second year of pilot-scale study, respectively.

Table S6. Core and rare bacterial population observed in full-scale AS-WWTPs in family level.

**Phylum**	**class**	**order**	**family**	**% of read**
**Core families observed in all AS-WWTPs**				
Bacteroidetes	*Flavobacteriia*	*Flavobacteriales*	*Flavobacteriaceae*	8.7
	Sphingobacteriia	*Sphingobacteriales*	N.A	1.1
			Flexibacteraceae	1.5
Chloroflexi	*Anaerolineae*	*Anaerolineales*	*Anaerolinaceae*	1.8
Firmicutes	*Bacilli*	*Lactobacillales*	*Carnobacteriaceae*	1.2
Proteobacteria	*Alphaproteobacteria*	*Sphingomonadales*	*Sphingomonadaceae*	2.1
	Betaproteobacteria	*Burkholderiales*	*Comamonadaceae*	8.0
	Betaproteobacteria	*Rhodocyclales*	*Rhodocyclaceae*	1.2
	Gammaproteobacteria	*Xanthomonadales*	*Sinobacteraceae*	0.5
TM7	*TM7-1*	N.A[a]	N.A	3.5
**Core families observed at least in 6 AS-WWTPs and with more than 1000 sequences**				
Acidobacteria	*Chloracidobacteria*	N.A	N.A	3.3
Actinobacteria	*Actinobacteria*	*Actinomycetales*	*Intrasporangiaceae*	1.5
Bacteroidetes	*Bacteroidia*	*Bacteroidales*	N.A	1.3
	Sphingobacteriia	*Sphingobacteriales*	*Chitinophagaceae*	4.1
			Saprospiraceae	6.8
Chloroflexi	*Anaerolineae*	*Caldilineales*	*Caldilineaceae*	1.8
		envOPS12	N.A	1.6
Proteobacteria	*Alphaproteobacteria*	*Rhizobiales*	*Hyphomicrobiaceae*	1.2
		Rhodobacterales	*Rhodobacteraceae*	2.2
	Gammaproteobacteria	*Pseudomonadales*	*Moraxellaceae*	1.3
		Xanthomonadales	*Xanthomonadaceae*	2.4
TM7	*TM7-3*	*I025*	N.A	1.8
		N.A	N.A	1.7
Verrucomicrobia	*Verrucomicrobiae*	*Verrucomicrobiales*	*Verrucomicrobiaceae*	2.4
**Plant-specific observed in ≤ 50% of AS-WWTPs and abundant (>6% of reads from plant)**				
Acidobacteria	*Acidobacteria-6*	*iii1-15*	*mb2424*	0.6
Actinobacteria	*Acidimicrobiia*	*Acidimicrobiales*	*C111*	0.3
	Actinobacteria	*Actinomycetales*	*Gordoniaceae*	0.4
			Microbacteriaceae	0.3
Chlorobi	*SJA-28*	N.A	N.A	0.3
Chloroflexi	*Anaerolineae*	*OP11*	N.A	0.5
	Chloroflexi	*Roseiflexales*	*Kouleothrixaceae*	0.3
Fusobacteria	*Fusobacteria*	*Fusobacteriales*	*Leptotrichiaceae*	1.0
OP11	*OP11-3*	N.A	N.A	0.4
Planctomycetes	*Planctomycetia*	*Gemmatales*	*Gemmataceae*	0.3
Proteobacteria	*Alphaproteobacteria*	*Rhizobiales*	*Bradyrhizobiaceae*	0.9
	Betaproteobacteria	*Methylophilales*	*Methylophilaceae*	0.4
	Deltaproteobacteria	*Myxococcales*	N.A	0.3
	Gammaproteobacteria	*Thiotrichales*	*Thiotrichaceae*	0.6
TM7	*TM7-3*	N.A	N.A	0.3

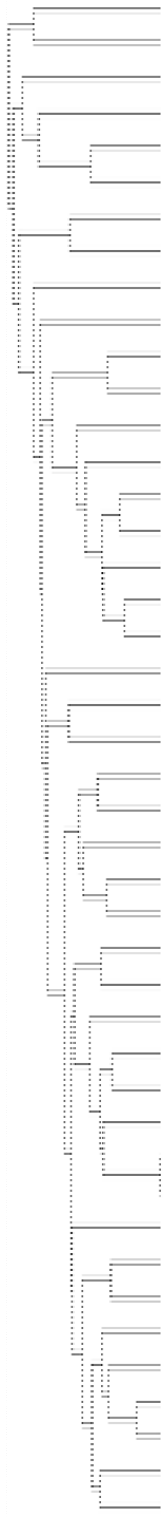

Table S7. Observed abundant families in LaPrairie-WWTP reactors.

**Phylum**	**Class**	**Order**	**Family**
Proteobacteria	*Deltaproteobacteria*	*Myxococcales*	*xHaliangiaceae*
Proteobacteria	*Gammaproteobacteria*	*Thiotrichales*	*Thiotrichaceae*
OP11	*WCHB1-64*	*d153*	N.A[a]
TM7	*TM7-3*	*I025*	N.A
Actinobacteria	*Actinobacteria*	*Actinomycetales*	*Intrasporangiaceae*
TM7	*TM7-3*	N.A	N.A
Proteobacteria	*Alphaproteobacteria*	*Rhodospirillales*	N.A
Verrucomicrobia	*[Pedosphaerae]*	*[Pedosphaerales]*	*auto67_4W*
Proteobacteria	*Deltaproteobacteria*	*Myxococcales*	*Polyangiaceae*
Proteobacteria	*Deltaproteobacteria*	*Myxococcales*	*Other*
Chloroflexi	*Anaerolineae*	*envOPS12*	N.A
Chloroflexi	*Anaerolineae*	*SR11*	*A4b*
Proteobacteria	*Gammaproteobacteria*	*Xanthomonadales*	*Xanthomonadaceae*
Bacteroidetes	*Sphingobacteriia*	*Sphingobacteriales*	*Saprospiraceae*
Proteobacteria	*Betaproteobacteria*	*Burkholderiales*	*Comamonadaceae*
Proteobacteria	*Betaproteobacteria*	*Methylophilales*	*Methylophilaceae*
Bacteroidetes	*Flavobacteriia*	*Flavobacteriales*	*Flavobacteriaceae*
Bacteroidetes	*Sphingobacteriia*	*Sphingobacteriales*	*Chitinophagaceae*
Verrucomicrobia	*Verrucomicrobiae*	*Verrucomicrobiales*	*Verrucomicrobiaceae*
Proteobacteria	*Gammaproteobacteria*	*Pseudomonadales*	*Moraxellaceae*
Acidobacteria	*Chloracidobacteria*	N.A	N.A
Bacteria	*TM7*	N.A	N.A
Acidobacteria	*Acidobacteria-6*	*iii1-15*	*mb2424*
Proteobacteria	*Alphaproteobacteria*	N.A	N.A
Proteobacteria	*Betaproteobacteria*	*YCC11*	N.A
Verrucomicrobia	*[Spartobacteria]*	*Chthoniobacterales*	*[Chthoniobacteraceae]*
Proteobacteria	*Alphaproteobacteria*	*Sphingomonadales*	*Sphingomonadaceae*
Proteobacteria	*Betaproteobacteria*	*Rhodocyclales*	*Rhodocyclaceae*
SR1	N.A	N.A	N.A
Sphingobacteriia	*Sphingobacteriales*	*Sphingobacteriales*	*Sphingobacteria*
N.A	N.A	N.A	N.A
Chloroflexi	*Anaerolineae*	*Caldilineales*	*Caldilineaceae*
Proteobacteria	*Alphaproteobacteria*	*Sphingomonadales*	N.A
Proteobacteria	*Deltaproteobacteria*	*Myxococcales*	N.A
Proteobacteria	*Deltaproteobacteria*	*Myxococcales*	N.A
Chloroflexi	*Anaerolineae*	*OPB11*	N.A
Proteobacteria	*Alphaproteobacteria*	*Rhodobacterales*	*Rhodobacteraceae*
Proteobacteria	*Alphaproteobacteria*	*Rhizobiales*	*Hyphomicrobiaceae*
OP8	*OP8_1*	*OPB95*	N.A
Chloroflexi	*Anaerolineae*	*Anaerolineales*	*Anaerolinaceae*
Planctomycetes	*Planctomycetia*	*Pirellulales*	*Pirellulaceae*
Planctomycetes	*Planctomycetia*	*Planctomycetales*	*Planctomycetaceae*
OP11	*OP11-3*	N.A	N.A
TM7	*TM7-1*	N.A	N.A

a: N.A: not assigned

a: N.A: not assigned

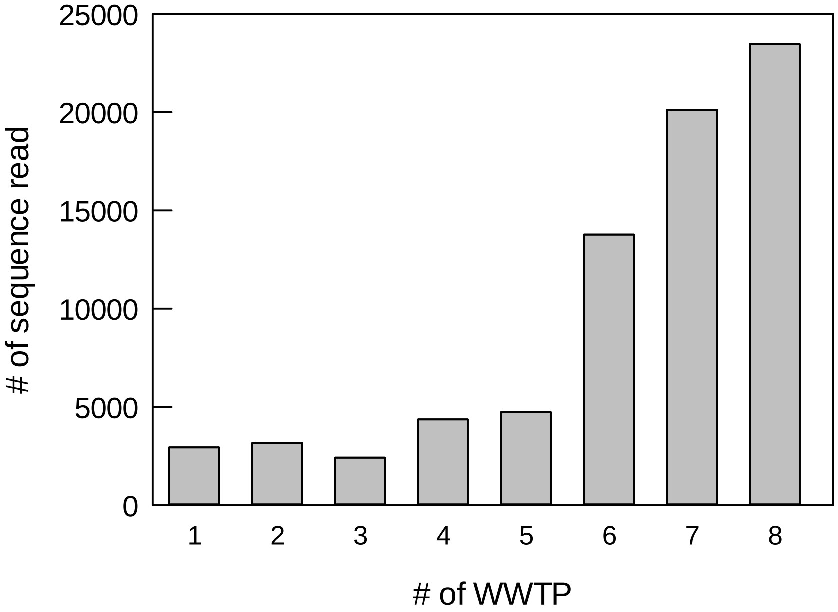

Fig. S6. Abundance of shared sequences in WWTPs.

References:

Caporaso, J.G., Bittinger, K., Bushman, F.D., DeSantis, T.Z., Andersen, G.L. and Knight, R. (2010) PyNAST: a flexible tool for aligning sequences to a template alignment. *Bioinformatics* **26**, 266-267.

DeSantis, T.Z., Hugenholtz, P., Larsen, N., Rojas, M., Brodie, E.L., Keller, K., Huber, T., Dalevi, D., Hu, P. and Andersen, G.L. (2006) Greengenes, a chimera-checked 16S rRNA gene database and workbench compatible with ARB. *Appl Environ Microbiol* **72**, 5069-5072.

Edgar, R.C. (2010) Search and clustering orders of magnitude faster than BLAST. *Bioinformatics* **26**, 2460-2461.

Lozupone, C., . and Knight, R., . (2005) UniFrac: a new phylogenetic method for comparing microbial communities. *Applied Environmnetal Microbiology* **71**, 8228-8235.

Pinto, A.J. and Raskin, L. (2012) PCR biases distort Bacterial and Archaeal community structure in Pyrosequencing datasets. *PLoS One* **7**, 1-16.

Wang, Q., Garrity, G.M., Tiedje, J.M. and Cole, J.R. (2007) Naive Bayesian classifier for rapid assignment of rRNA sequences into the new bacterial taxonomy. *Appl Environ Microbiol* **73**, 5261-5267.
